# Accurate estimation of modulation amplitude in Brillouin optical correlation-domain reflectometry based on Rayleigh noise spectrum

**DOI:** 10.1038/s41598-024-56426-2

**Published:** 2024-04-06

**Authors:** Keita Kikuchi, Heeyoung Lee, Ryo Inoue, Kouta Ozaki, Haruki Sasage, Yosuke Mizuno

**Affiliations:** 1 Graduate School of Engineering and Science, Shibaura Institute of Technology, Tokyo, 135-8548 Japan; 2 Faculty of Engineering, Yokohama National University, Yokohama, 240-8501 Japan; 3 Institute for Multidisciplinary Sciences, Yokohama National University, Yokohama, 240-8501 Japan

**Keywords:** Optical techniques, Electrical and electronic engineering

## Abstract

In Brillouin optical correlation-domain reflectometry (BOCDR), spatial resolution relies on the modulation amplitude of the light. We propose a Rayleigh-based method that utilizes the spectral width of Rayleigh-induced noise to measure this amplitude without altering the setup or requiring an optical spectrum analyzer. With high frequency resolution and ease of implementation, our approach enhances the convenience and accuracy of spatial resolution evaluation in BOCDR.

## Introduction

Brillouin-based optical fiber sensing has attracted substantial research attention due to its unique capability to measure temperature and strain distributions along a fiber under test (FUT)^1–5^. This feature is particularly valuable in applications such as structural health monitoring. Among the various approaches designed for distributed measurements, correlation-domain techniques are distinguished by their high spatial resolution and random accessibility to measurement points, setting them apart from traditional time-domain^6–9^ and frequency-domain methods^10,11^. Brillouin optical correlation-domain sensing encompasses two major configurations: Brillouin optical correlation-domain analysis (BOCDA)^12–17^, which leverages stimulated Brillouin scattering via the interaction of counter-propagating pump and probe lights with acoustic waves, and Brillouin optical correlation-domain reflectometry (BOCDR)^18–25^, which relies on spontaneous Brillouin scattering and allows single-end accessibility. This paper focuses specifically on the BOCDR technique.

The spatial resolution in BOCDR is critically determined by an inverse relationship with the product of the modulation amplitude and modulation frequency of the light^18,19^. Consequently, precision in measuring the modulation amplitude is pivotal for assessing spatial resolution. Traditional methods have required observation of the modulated light spectrum through an optical spectrum analyzer (OSA)^18^ or a separate heterodyne detection system with an electrical spectrum analyzer (ESA)^26^. Utilizing an OSA, though, is encumbered by limitations such as restricted frequency resolution, significant cost, and bulky size. Conversely, while a separate heterodyne detection system with an ESA offers high-frequency resolution, it demands alterations to the BOCDR system, detracting from user convenience^26^.

In this study, we propose a new method to measure the modulation amplitude without necessitating any alterations to the BOCDR experimental setup. By capitalizing on the spectral width of noise induced by Rayleigh scattering, our method not only enables modulation amplitude measurement without restrictions on the length of the FUT but also achieves high-frequency resolution. By employing the experimental setup of BOCDR with an ESA to directly observe Rayleigh-noise components, we ensure a straightforward implementation process, thus enhancing overall convenience. Note that this paper builds on preliminary findings presented in a prior conference paper^27^ and offers a more comprehensive and reasoned analysis, enriched by detailed experimental insights.

## Principle and proposal

**Figure 1:**
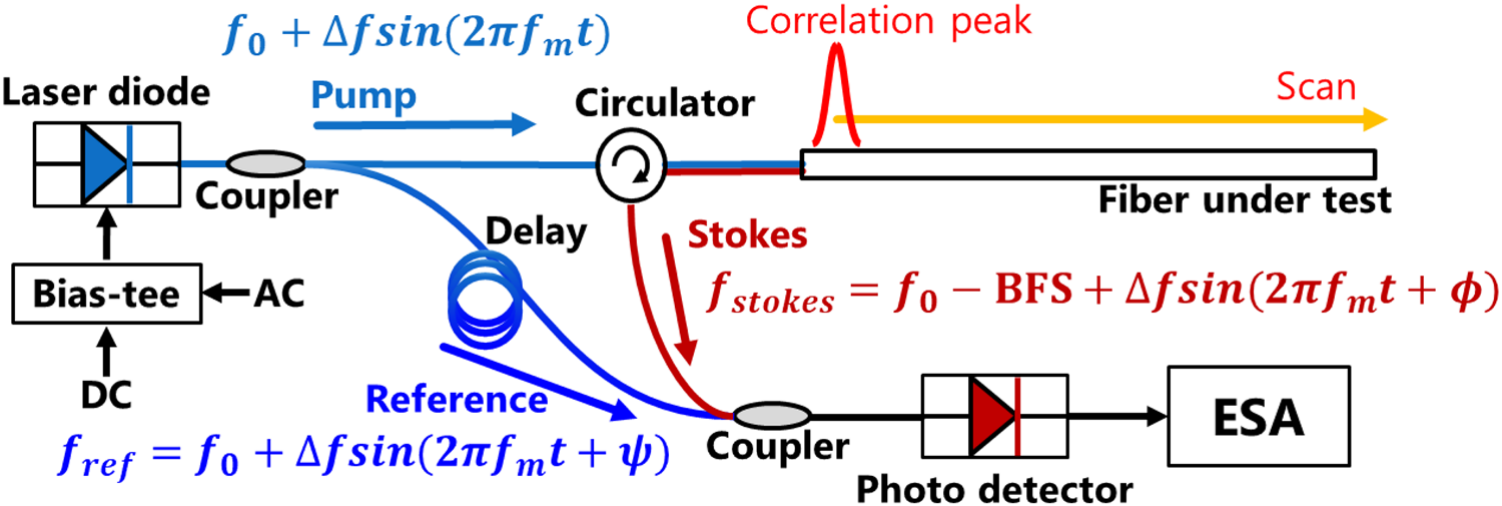
Conceptual setup of BOCDR. BFS: Brillouin frequency shift, ESA: electrical spectrum analyzer.

The schematic diagram of BOCDR is depicted in Fig. 1, illustrating the underlying principles^12,18^. Through the application of sinusoidal modulation to the incident light, “correlation peaks” emerge at specific positions along the FUT, where a strong temporal correlation exists between the Stokes and reference lights. By adjusting the modulation frequency, these correlation peaks are scanned along the FUT, enabling the acquisition of the Brillouin gain spectrum (BGS) at any desired location. The resulting distribution of the Brillouin frequency shift (BFS) can then be converted into a corresponding strain or temperature distribution, owing to the linear relationship between BFS and strain/temperature.

The spatial resolution $\Delta z$ and the measurement range ${d}_{{\text{m}}}$ (interval between the neighboring correlation peaks) of BOCDR are given by^18,19^:

1 $$\begin{array}{c}\Delta z=\frac{c\Delta {v}_{{\text{B}}}}{2n{\uppi f}_{{\text{m}}}\Delta f}\end{array} ,$$

2 $$\begin{array}{c}{d}_{{\text{m}}}=\frac{c}{2n{f}_{{\text{m}}}}\end{array} ,$$

where $c$ is the velocity of light, $\Delta {v}_{{\text{B}}}$ is the Brillouin bandwidth (~ 30 MHz) in optical fibers, $n$ is the refractive index of the fiber core, ${f}_{{\text{m}}}$ is the modulation frequency, and $\Delta f$ is the modulation amplitude. The sensing position *x* within the FUT can be expressed using the measurement range ${d}_{{\text{m}}}$, the order of the correlation peak *k*, and the location of the proximal end of the FUT relative to the 0th correlation peak *D*:

3 $$x=k\cdot {d}_{{\text{m}}}-D.$$

Here, *k* is determined by the relative optical path lengths between the reference and the Brillouin-scattered lights. Specifically, the 0th-order peak corresponds to points of identical path length. Orders above zero (*k* = 1, 2, …) indicate scattered light paths that exceed the reference path length by multiples of *d*_m_. Conversely, negative orders (*k* =  − 1, − 2, …) represent scattered light paths shorter than the reference path by *d*_m_ increments. The parameter *D* adopts a positive value when located further from, and a negative value when closer to, the 0th-order peak, mirroring the sign convention of *k*.

**Figure 2:**
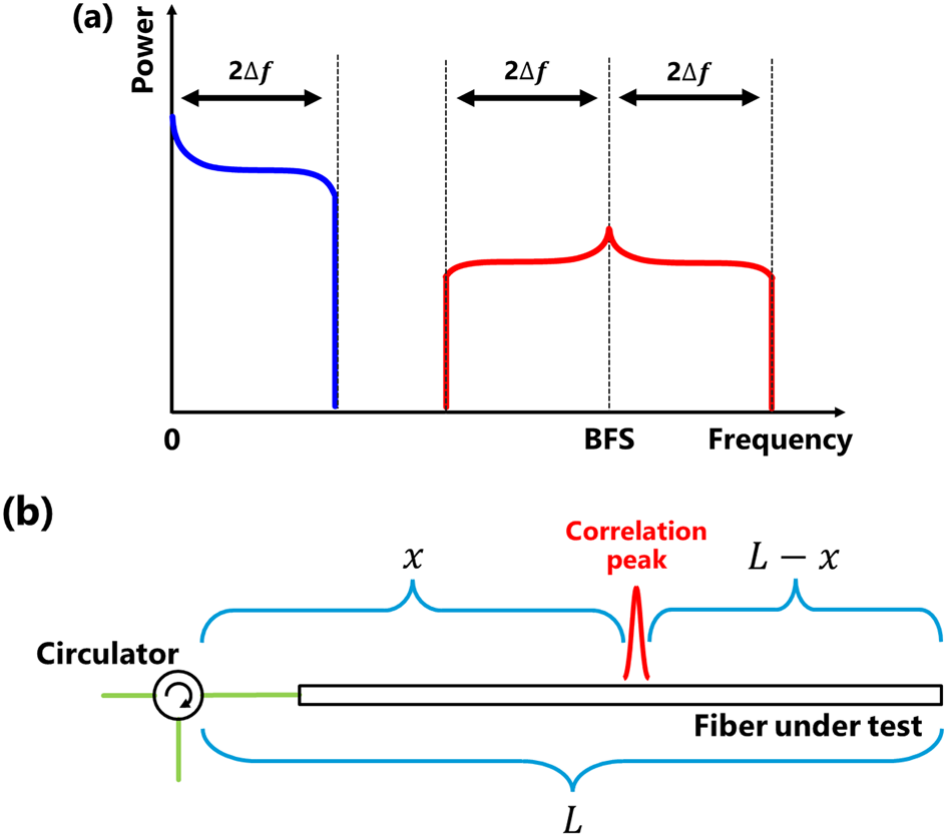
(**a**) Schematic electrical spectra of the BOCDR output when the FUT length exceeds half of the measurement range; *∆f* indicates the modulation amplitude. (**b**) Definitions of *L* and *x* in Eq. (4).

Figure 2a illustrates the schematic diagram of the noise spectrum caused by Rayleigh scattering, which is utilized for measuring the modulation amplitude. It has been reported that, when the length of the FUT exceeds half of the measurement range, the spectral width of the Rayleigh-induced noise observed on the ESA becomes twice the modulation amplitude^18,19^. Therefore, when the length of the FUT exceeds half of the measurement range, the modulation amplitude can be estimated as half of the noise width. However, when the length of the FUT is less than half of the measurement range, the compensation method has not been sufficiently investigated.

When the FUT length is shorter than half of the measurement range, if a correlation peak exists within the FUT (refer to Fig. 2b), the upper limit of the achievable modulation amplitude *∆f*_max_ has been reported to be^19^:

4 $$\Delta {f}_{max}=\frac{{v}_{{\text{B}}}}{2}\cdot {\text{cosec}}\left\{\frac{\uppi }{{d}_{{\text{m}}}}{{\max}}\left(L-x, x\right)\right\},$$

where ${v}_{{\text{B}}}$ is the BFS and *L* is the total length of the FUT. Equation (2) suffices for estimating the maximum modulation amplitude, but in practical applications, setting the modulation amplitude to its maximum can risk laser damage. Therefore, by substituting ${v}_{{\text{B}}}$ on the right side of Eq. (4) with the Rayleigh noise width ${W}_{{\text{R}}}$, we derive a formula that offers arbitrary modulation amplitude as a function of the Rayleigh width, as:

5 $$\Delta {f}_{{\text{R}}}=\frac{{W}_{{\text{R}}}}{2}\cdot {\text{cosec}}\left\{\frac{\uppi }{{d}_{{\text{m}}}}{\text{max}}\left(L-x, x\right)\right\},$$

where $\Delta {f}_{{\text{R}}}$ represents the estimated value of the modulation amplitude. By substituting Eqs. (2) and (3) into Eq. (5), the true modulation amplitude can be estimated from the Rayleigh noise width for any given modulation frequency:

6 $$\Delta {f}_{{\text{R}}}=\frac{{W}_{{\text{R}}}}{2}\cdot {\text{cosec}}\left\{\frac{\uppi }{{d}_{{\text{m}}}}{\text{max}}\left(L-k\cdot \frac{c}{2n{f}_{{\text{m}}}}+{\text{D}}, k\cdot \frac{c}{2n{f}_{{\text{m}}}}-D\right)\right\}.$$

On the other hand, our approach does not encompass scenarios where the length of the FUT is less than half of the measurement range, and no correlation peak is present within the FUT. This specific case results in low-power Rayleigh noise, offering no tangible advantages for estimating the modulation amplitude and therefore lies outside the scope of our method.

## Method

To substantiate the efficacy of our method in estimating the true modulation amplitude based on the Rayleigh noise width, we conducted demonstrations under two specific conditions: (i) the FUT length exceeds half of the measurement range, with no requirement for the presence of correlation peaks within the FUT, and (ii) the FUT length is less than half of the measurement range, with a correlation peak present within the FUT. A third scenario, (iii) where the FUT length is shorter than half of the measurement range and lacks a correlation peak within the FUT, was intentionally excluded from our consideration.

**Figure 3:**
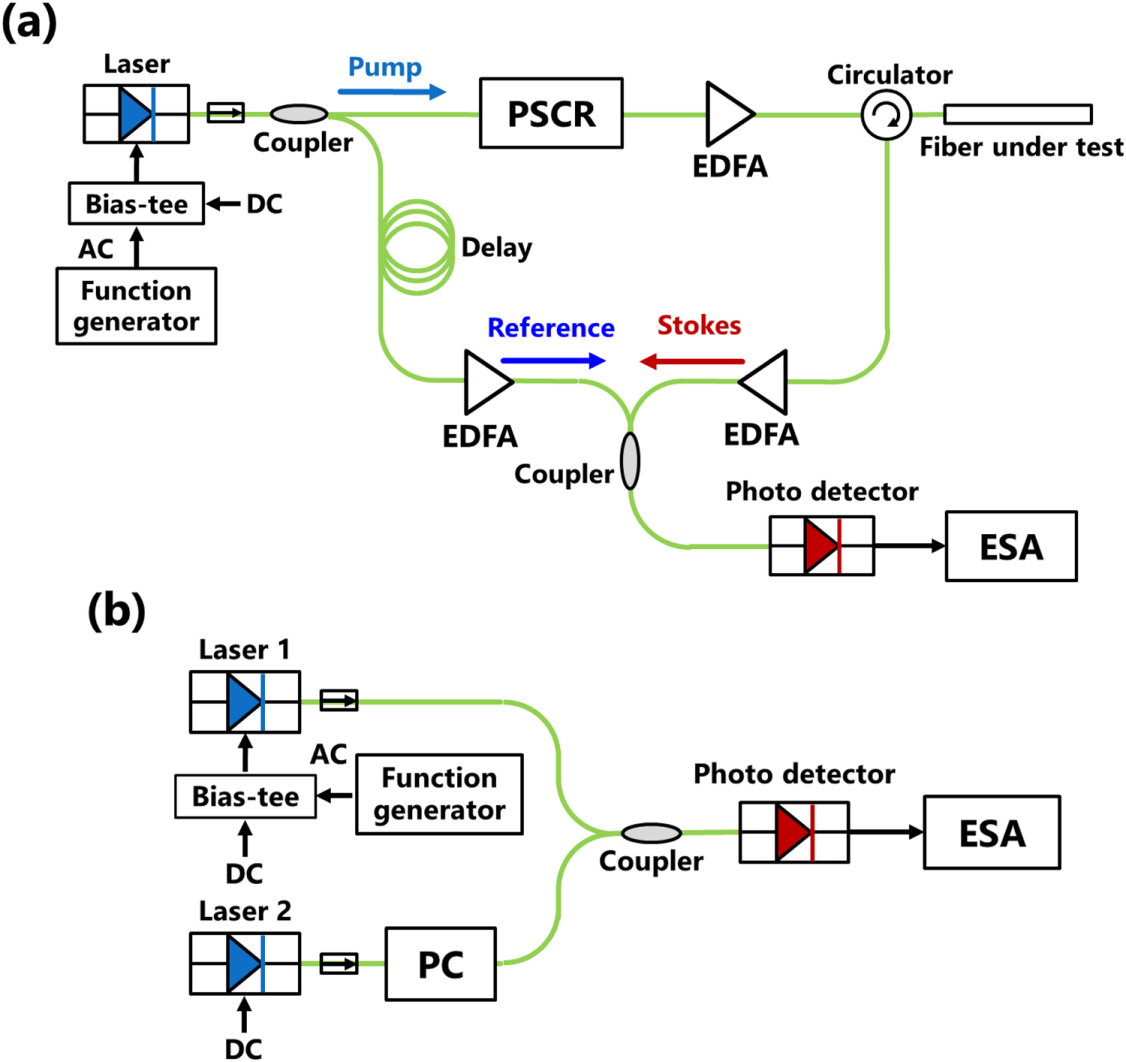
(**a**) Experimental setup of BOCDR. EDFA: erbium-doped fiber amplifier, ESA: electrical spectrum analyzer, PSCR: polarization scrambler. (**b**) Experimental setup for measuring the true modulation amplitude. PC: polarization controller.

Figure 3a illustrates the experimental setup of the BOCDR system used to measure the Rayleigh noise width, which is basically the same as the standard setup^18^. A distributed-feedback (DFB) laser at 1550 nm was employed as the light source. The total length of the FUT, including a circulator, was 21.0 m. Erbium-doped fiber amplifiers (EDFAs) were used to amplify the output power of the pump light up to 24 dBm and the Stokes light up to 5 dBm, while the reference light was amplified up to 4.5 dBm. A 140-m-long delay fiber was inserted in the reference path. Subsequently, the experimental setup for measuring true modulation amplitude^26^ is depicted in Fig. 3b. Laser 1 is the same as that used in the BOCDR system, and Laser 2 (same type of DFB laser) at 1550 nm was adjusted to have a center frequency difference of approximately 6 GHz, by controlling the driving current. For all measurements, the resolution bandwidth, video bandwidth, and sweep time of the ESA were 3 MHz, 1 kHz, and 60 ms, respectively, and 40 times averaging was performed. The voltage amplitude of the function generator (FG) was fixed at 1.0 V_p-p_, and measurements were performed by varying the modulation frequency within the range of 1.6–3.0 MHz with a step size of 0.1 MHz. Based on these experimental conditions, the modulation frequencies of ~ 2.42 MHz or higher corresponded to (i), while the modulation frequencies ranging from ~ 1.60 MHz to ~ 2.42 MHz corresponded to (ii). Note that the modulation frequencies of ~ 1.60 MHz or lower pertained to (iii), a case that was explicitly excluded from consideration in this experiment. Considering the negative order of the correlation peak (*k* = −1) in Eq. (6), the *D* value was determined as −63.7 m.

## Results

**Figure 4:**
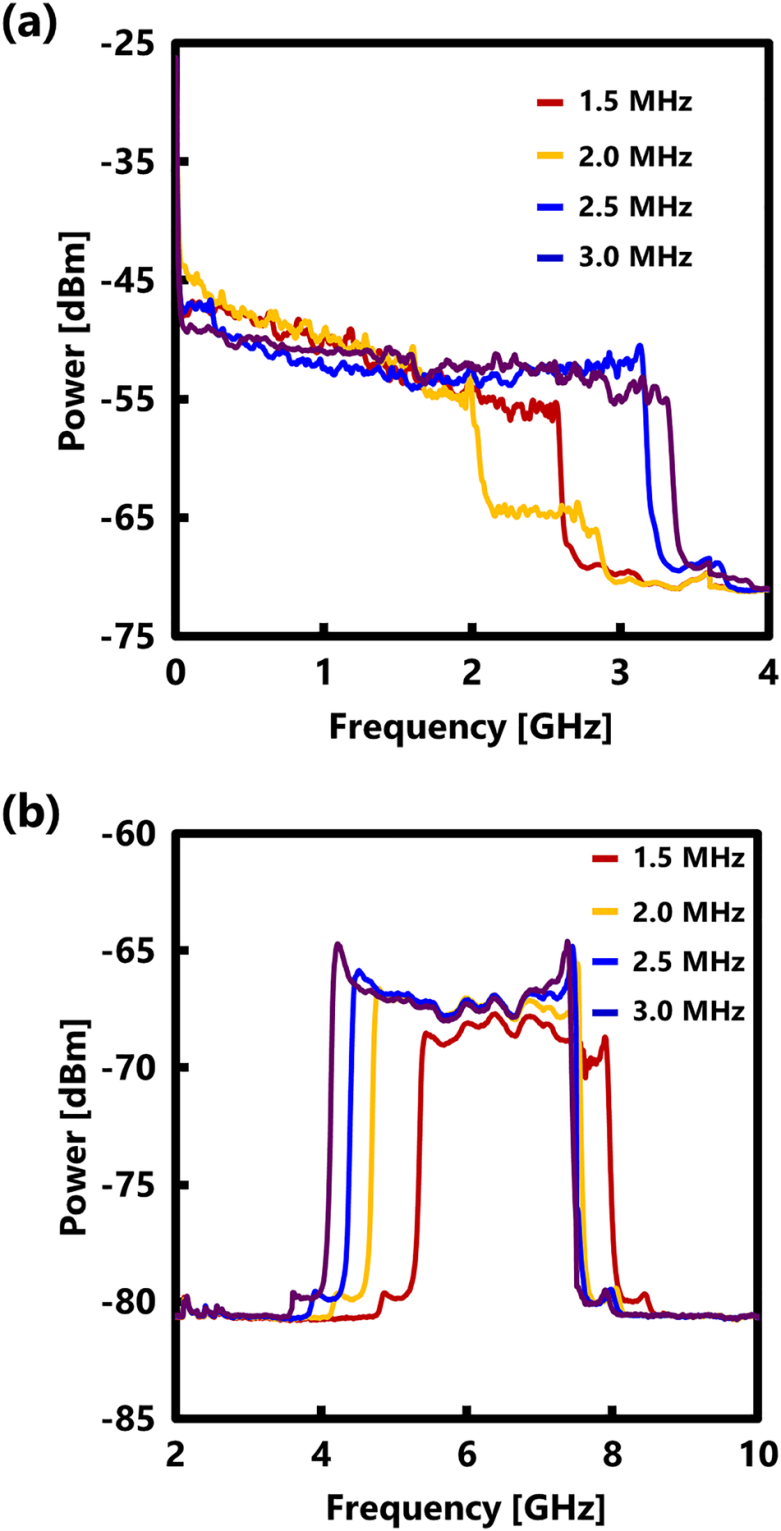
Measured electrical spectra (at four modulation frequencies), used to measure (**a**) the noise width and (**b**) true modulation amplitude.

**Figure 5:**
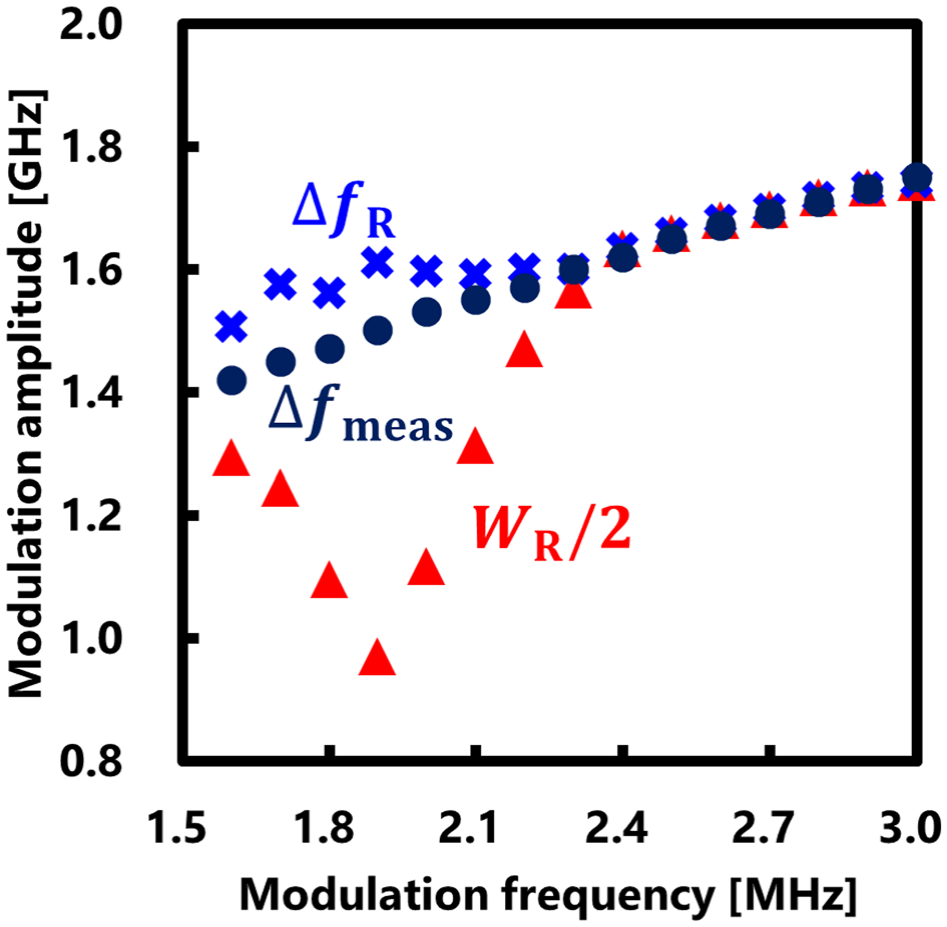
Measured modulation frequency dependences of half the Rayleigh noise width ${W}_{{\text{R}}}/2$, the estimated modulation amplitude $\Delta {f}_{{\text{R}}}$, and the true modulation amplitude $\Delta {f}_{{\text{meas}}}$.

Figure 4a illustrates the Rayleigh noise spectrum obtained using the BOCDR system, while Fig. 4b displays the interference spectrum used for measuring the true modulation amplitude. The Rayleigh noise width exhibited a narrowing trend initially as the modulation frequency increased, followed by a widening trend, whereas the true modulation amplitude consistently increased. This indicates that the method for estimating the true modulation amplitude from the noise width differs depending on conditions (i) and (ii). Figure 5 presents the modulation frequency dependencies of half the value of the Rayleigh noise width ${W}_{{\text{R}}}/2$, the estimated modulation amplitude $\Delta {f}_{{\text{R}}}$ calculated using the Rayleigh noise, and the measured true modulation amplitude $\Delta {f}_{{\text{meas}}}$. Notably, around 1.9 MHz, where the correlation peak existed within the FUT and the FUT length was shorter than half of the measurement range, the Rayleigh noise width significantly deviated from the true modulation amplitude. Conversely, the estimated $\Delta {f}_{{\text{R}}}$ values calculated from the Rayleigh noise width closely aligned with the true modulation amplitude, with a maximal error of ~ 9% at 1.7 MHz. These experimental results demonstrate the effectiveness of our method for estimating the modulation amplitude from the measured Rayleigh noise width, irrespective of the FUT length.

## Conclusion

This study presented a new method for estimating the modulation amplitude in BOCDR without requiring modifications to the experimental setup or the use of an OSA. By exploiting the spectral width of the Rayleigh-induced noise, our method enables accurate measurement of the modulation amplitude while overcoming length restrictions on the FUT. With its high frequency resolution and easy implementation, we believe that this approach will enhance the convenience of modulation amplitude measurement, thereby facilitating spatial resolution evaluation in BOCDR.

## Data Availability

Data underlying the results presented in this paper are not publicly available at this time but may be obtained from authors upon reasonable request by contacting Yosuke Mizuno at mizuno-yosuke-rg@ynu.ac.jp.
